# First Report of *Meloidogyne Incognita* Infecting *Mitragyna Speciosa* in the United States

**DOI:** 10.2478/jofnem-2022-0021

**Published:** 2022-07-02

**Authors:** Alemayehu Habteweld, Wade Davidson, Johan Desaeger, William T. Crow

**Affiliations:** 1 Entomology and Nematology Department, University of Florida, Gainesville, FL 32611 U.S.A; 2 Gulf Coast Research and Education Center, University of Florida, Wimauma, FL 33598 U.S.A

**Keywords:** Diagnosis, Florida, kratom, mitochondrial haplotyping, root galls, root-knot nematode, species-specific primers

## Abstract

Kratom (*Mitragyna speciosa*) belongs to the coffee family of Rubiaceae. The tree is native to Southeast Asia and primarily grown in Malaysia, Thailand, and Indonesia. Recently, it has been introduced and cultivated in other countries including the United States. The leaves and extracts of the leaves are used for medicinal and recreational purposes. In February 2022, kratom root and soil samples were submitted to the University of Florida Nematode Assay Laboratory for diagnosis by a commercial grower in Florida. Root galls were observed on the roots. On examination of soil and root samples, it is revealed that high numbers of root-knot nematodes (*Meloidogyne* sp.) are present. Molecular species identification was performed by a combination of the mitochondria haplotyping and species-specific primer techniques using TRNAH/MHR106 and MORF/MTHIS primer sets and *Meloidogyne incognita*-specific primers (MIF/MIR). The root-knot nematode infecting kratom is identified as *M. incognita* by molecular analysis. To our knowledge, this paper is the first report of *M. incognita* infecting kratom in the United States.

Kratom (*Mitragyna speciosa*) is a tropical evergreen tree that belongs to the family of Rubiaceae, which is native to the Southeast Asia. Its leaves contain two psychoactive compounds, mitragynine and 7-hydroxymytragynine, that produce either sedative or stimulative effects, depending on the dose. While kratom is currently legal in most U.S. States, it is illegal in Alabama, Arkansas, Indiana, Rhode Island, Vermont, and Wisconsin. Additionally, the U.S. Food and Drug Administration warns consumers against using kratom products and the U.S. Drug Enforcement Agency lists it as a drug of chemical concern (DEA, 2020). With the exception of Sarasota County, kratom is currently legal in the state of Florida (McCurdy *et al*., 2020).

Fresh kratom leaves were chewed to increase work efficiency and relieve fatigue for manual laborers in Southeast Asia (Suwanlert, 1975). Fresh and dried leaves were also brewed into teas in Malaysia and Thailand for a range of ailments including diabetes, diarrhea, fever, pain, and for use as a wound poultice (Ahmad and Aziz, 2012; McCurdy *et al*., 2020). There are also reports that kratom has been used as a substitute for opium, and to reduce opioid withdrawal symptoms. In the United States, kratom is often formulated into capsules and sold in stores (McCurdy *et al*., 2020).

In February 2022, kratom root and soil samples were submitted to the University of Florida Nematode Assay Laboratory (NAL) for diagnosis by a commercial grower in Florida. Extensive galling on the kratom roots was evident (Fig. 1A), and female root-knot nematodes were observed within these galls (Fig. 1B). Examination of soil and root samples also revealed the presence of high numbers of root-knot nematodes (*Meloidogyne* sp.). Since there are no records of infections of root-knot nematodes on kratom in the United States, a study was conducted to identify the root-knot nematode species extracted from the kratom root samples using a combination of the mitochondria haplotyping and species-specific primer techniques.

**Figure 1:**
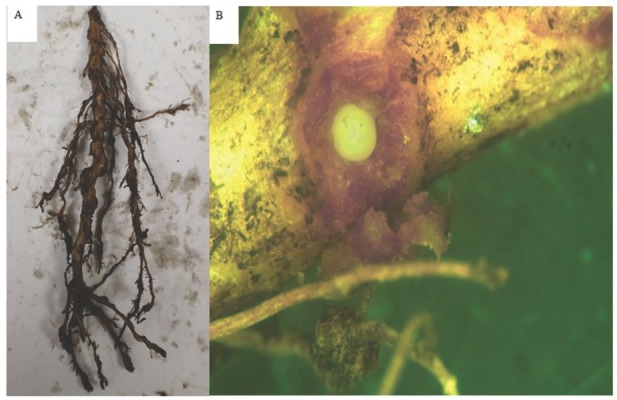
(A) Galled kratom (*Mitragyna speciosa*) root sample received from Hardee County, Florida; (B) a root-knot nematode female inside the kratom root sample.

## Materials and Methods

### Nematode population and extraction

Eleven mixed soil and root samples were submitted to NAL for nematode diagnosis in February 2022 by a commercial kratom grower in Hardee County in Florida. From these samples, plant-parasitic nematodes were extracted from 100 cm^3^ of soil by centrifugal-flotation (Jenkins, 1964) and from 10 g of roots using a modified mist extraction (Crow *et al*., 2020). All plant-parasitic nematodes found in the soil and root samples were identified at the genus level and counted.

### Molecular species identification of a root-knot nematode population infecting kratom

Two individual female *Meloidogyne* sp. were collected from the infected roots (Fig. 1B) for genomic DNA extraction. The total genomic DNA was extracted following the Proteinase K method as described in Pagan *et al*. (2015). Each single female was transferred to DNA extraction buffer containing 18 **m**l Tris-EDTA buffer, 1 **m**l of 2% triton, and 1 **m**l of Proteinase K (20 mg/ ml) in a PCR tube. The nematode in the extraction buffer was frozen and thawed three times and placed at **-**20**°**C overnight. The DNA was extracted by incubating the tube at 56**°**C for 1 hr followed by deactivation at 95**°**C for 15 min using T100^TM^ thermo cycler (Bio-Rad Laboratories, Inc., Hercules, CA).

Mitochondrial haplotyping and species-specific primer techniques, which are the routine procedures used in NAL for root-knot nematodes identification, were performed. Two mitochondrial DNA regions span the intergenic spacer and part of the adjacent large subunit ribosomal RNA gene (lrDNA) using TRNAH/MHR106 and MORF/MTHIS primer sets. TRNAH/MHR106 product span the tRNA^His^ and lrDNA region, and MORF/ MTHIS product span the intergenic spacer and tRNA^His^ region (Pagan *et al*., 2015; Stanton *et al*., 1997). DNA amplification was performed in 25 **m**l of reaction mix containing 12.5 **m**l 2**⨯** Hot Start Master mix (Genessee Scientific, San Diego, CA), 1.25 **m**l forward and reverse primers, 8.5 **m**l sterile nuclease free water (Thermo Fisher Scientific, Gainesville, FL), and 1.5 **m**l template DNA. The thermocycling reaction using TRNAH/MHR106 and MORF/MTHIS primer sets were as follows: 95**°**C for 15 min, followed by 35 cycles of 94**°**C for 1 min, 50**°**C for 1 min, and 68**°**C for 1 min, and a final extension step of 68**°**C for 5 min (Pagan *et al*., 2015). To determine the mitochondrial haplotype, the amplified fragments were separated by electrophoresis in 1XTAE buffer and 1.5% agarose gel, stained with SYBR safe DNA Gel Stain (Invitrogen, Thermo Scientific Inc.) and visualized, and photographed under UV light using the ChemiDOC XRS One 4.5.2 program (Bio-Rad Laboratories, Life Science Group, Hercules, CA).

After the mitochondrial haplotype was determined, *Meloidogyne incognita*-specific (MIF/MIR) and *M. javanica*-specific (Fjav/Rjav) primers were used to confirm the root-knot nematode species (Adam *et al*., 2007). The thermocycling conditions for the species-specific primers were performed according to Adam *et al*. (2007). Once the identity of the species was confirmed, the mitochondrial genome spanning the gene encoding cytochrome oxidase II (COII), the intergenic spacer, tRNA^His^, and the large subunit of the ribosomal RNA gene (lrRNA) was amplified using C2F3/1108 primers (Powers and Harris, 1993; Powers *et al*., 2005). PCR clean-up and sequencing of the amplified product of C2F3/1108 were performed by Azenta US, Inc. (South Plainfield, New Jersey). The raw sequences were checked and edited manually using Geneious Prime 2022.1 (Auckland, New Zealand). The consensus sequence obtained was compared with those deposited in the GenBank database using BLAST engine search for sequence homology. The newly obtained consensus sequence was submitted to the GenBank database under accession number ON528104.

## Results and Discussion

Plant-parasitic nematode genera recovered from soil included large numbers of *Meloidogyne* (ranging from 100 to 637 second stage juveniles (J2)/100 cm^3^ of soil), along with *Belonolaimus* (ranging from 8 to 15 individuals/100 cm^3^ of soil), *Helicotylenchus* (ranging from 15 to 226 individuals/100 cm^3^ of soil), *Mesocriconema* (ranging from 6 to 226 individuals/100 cm^3^ of soil), *Hemicriconemoides* (ranging from 13 to 80 individuals/100 cm^3^ of soil), *Hemicaloosia* (ranging from 2 to 7 individuals/100 cm^3^ of soil), and *Xiphinema* (ranging from 1 to 3 individuals/100 cm^3^ of soil). Plant-parasitic nematode genera recovered from roots were *Meloidogyne* (ranging from 9 to 124 J2/10 g of root) and small numbers of *Pratylenchus* (ranging from 2 to 6 individuals/10 g of root).

Based on the mitochondria DNA analysis, the root-knot nematode population extracted from kratom root in the present study was identified as *M. incognita*. Identification of a closely related, asexually reproducing (apomictic) species complex including *M. incognita*, *M. javanica*, and *M. arenaria* is difficult using ribosomal RNA (rDNA). This is because sequence differences of rDNA copies within an individual RKN showed greater diversity than between species (Pagan *et al*., 2015). Due to its uniparental inheritance, the mitochondrial genome can circumvent some of the difficulties of differentiating these species (Powers and Harris, 1993; Stanton *et al*., 1997). Previous studies supported that the mitochondrial genome of the apomictic RKNs has revealed a useful source of diagnostic markers (Gissi *et al*., 2008; Powers *et al*., 2005). Later, a diagnostic tool based on the amplification of two mitochondrial regions that together span the intergenic spacer and part of the adjacent large subunit (16S) rRNA (lrDNA) was developed (Pagan *et al*., 2015). In this method, mitochondrial haplotypes can be assigned based on the size of the intergenic spacer and sequence polymorphism in the lrDNA.

**Figure 2:**
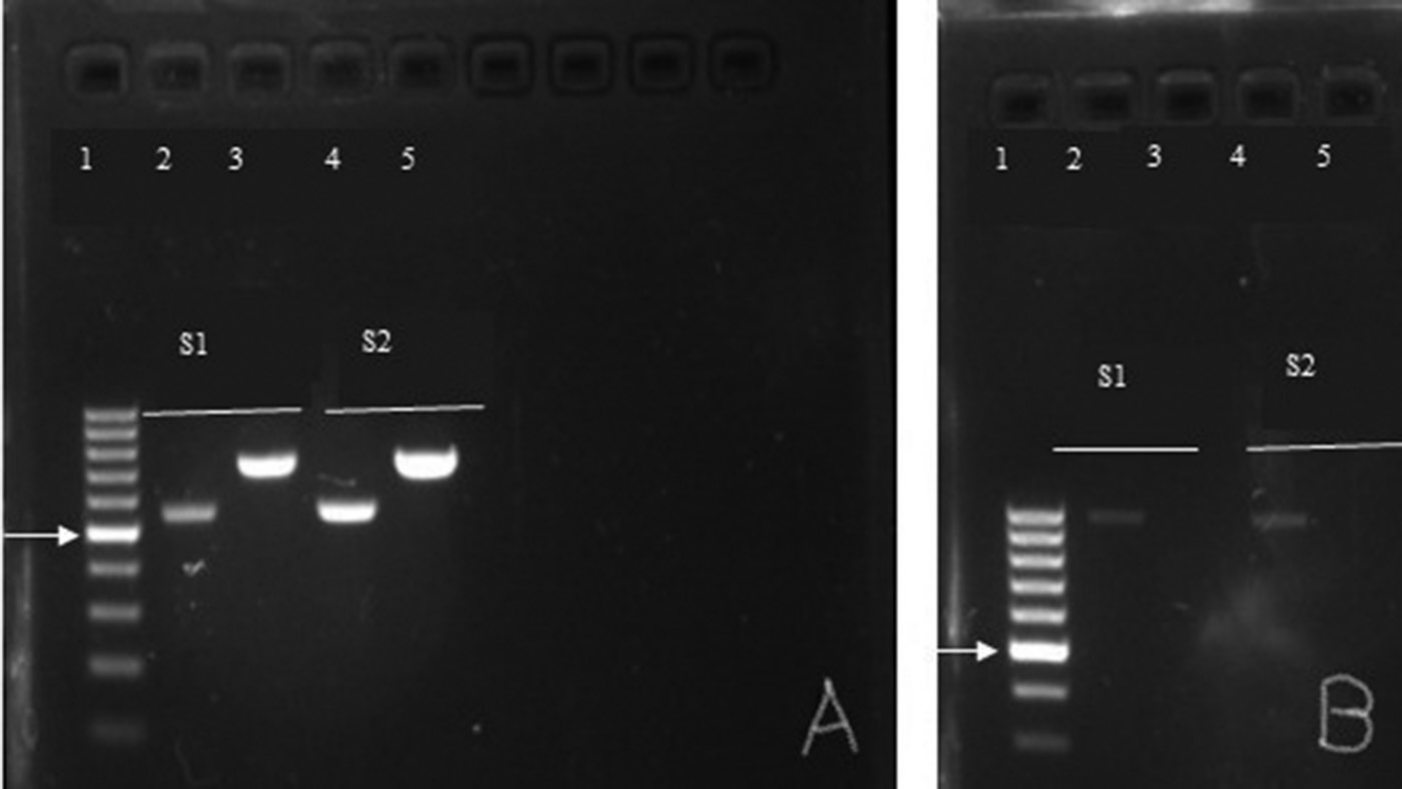
(A) Two mitochondrial DNA regions span the intergenic spacer and part of the adjacent large subunit ribosomal RNA gene (lrDNA) amplified by TRNAH/MHR106 (Lanes 2 and 4, 557-bp) and MORF/MTHIS (Lanes 3 and 5, 742-bp) primer sets for sample 1 (S1) and sample 2 (S2); (B) amplified products using *M. incognita*-specific (MIF/MIR) (Lanes 2 and 4) and *M. javanica-*specific (Fjav/Rjav) (Lanes 3 and 5) primers. Only MIF/MIR primers yielded an amplified product approximately 999-bp which indicates the root-knot nematode that infected kratom was *M. incognita*. No product was observed from Fjav/Rjav primers. Lane 1 **=** 1 kb DNA ladder, with the position of the 500-bp band indicated by the arrow.

The results of the present study showed that the sizes of the amplified products were approximately 557-bp and 742-bp for TRNAH/MHR106 and MORF/MTHIS primer sets, respectively (Fig. 2A). This mitochondrial haplotype could either be for *M. incognita* or for *M. javanica*. To differentiate between the two species, the species-specific primers MIF/MIR (*M. incognita*) and Fjav/Rjav (*M. javanica*) primer sets were used (Adam *et al*., 2007). Amplification using the *M. incognita*-specific primers yielded approximately 999-bp while no product was amplified from *M. javanica*-specific primers (Fig. 2B). This confirms that the root-knot nematode that infected the kratom roots was *M. incognita*. The amplified product of C2F3/1108 primers yielded approximately 1562-bp product and the sequence showed 99% identity (99% query cover) to several *M. incognita* sequences in the NCBI database such as LC547506 and FJ159614 from South Korea and France, respectively. To our knowledge, this is the first report of *M. incognita* infecting kratom in the United States.
